# Multi-Sensor Approach for the Monitoring of Halitosis Treatment via *Lactobacillus brevis* (CD2)—Containing Lozenges—A Randomized, Double-Blind Placebo-Controlled Clinical Trial

**DOI:** 10.3390/s150819583

**Published:** 2015-08-10

**Authors:** Enrico Marchetti, Simona Tecco, Marco Santonico, Chiara Vernile, Daniele Ciciarelli, Ester Tarantino, Giuseppe Marzo, Giorgio Pennazza

**Affiliations:** 1 Department MESVA, University of L’Aquila, Via Vetoio ed. Delta 6, 67100 L’Aquila, Italy; E-Mails: daky87@hotmail.it (D.C.), ester88x@hotmail.it (E.T.); giuseppe.marzo@cc.univaq.it (G.M.); 2 University Vita-Salute San Raffaele, I.R.R.C.S. San Raffaele Hospital, 20100 Milano, Italy; E-Mail: tecco.simona@hsr.it; 3 Center for Integrated Research—CIR, Unit of Electronics for Sensor Systems, “Università Campus Bio-Medico di Roma”, Via Alvaro del Portillo 21, 00128 Rome, Italy; E-Mails: m.santonico@unicampus.it (M.S.); c.vernile@unicampus.it (C.V.); g.pennazza@unicampus.it (G.P.)

**Keywords:** halitosis, bad breath, electronic nose, chemical sensors, sensor array, probiotics

## Abstract

The aim of this randomized clinical trial was to evaluate whether a recently described multi-sensor approach called BIONOTE^®^ is accurate enough to verify the efficacy of treatment of patients with halitosis. A treatment with *Lactobacillus brevis* (CD2)–containing lozenges, compared with placebo was tested. The BIONOTE^®^ was compared with traditional techniques used to detect halitosis: OralChroma™ and two calibrated odor judges enrolled for the organoleptic assessments. Twenty patients (10 treated and 10 placebo), suffering from active phase halitosis were included in the study. Treatment consisted of *Lactobacillus brevis* (CD2)—containing lozenges or placebo, 4 tablets/day for 14 days. t_0_ was before the beginning of the study; t_1_ was day 7 and t_2_ was day 14. The effectiveness of treatment was assessed through: (1) Rosenberg score; (2) Winkel tongue coating index (WTCI) anterior and posterior; (2) OralChroma™; (3) the new developed multi-sensor approach, called BIONOTE^®^ (test technique). Only the WTCI anterior revealed statistically significant changes between t_0_ and t_2_ data (*p* = 0.014) in the treated group. Except for the WTCI anterior, all diagnostic methods revealed the lack of effectiveness for halitosis of a 14-days treatment with *Lactobacillus brevis* (CD2)–containing lozenges. The BIONOTE^®^ multisensor system seems accurate in addition to OralChroma™ to assess the initial condition of halitosis and its mitigation during treatment.

## 1. Introduction

Non-selective gas sensor arrays are widely used, in a research context, for medical applications oriented to non-invasive prognosis, diagnosis and follow-up [1]. Despite promising results they cannot be used so far in the clinical practice [2]. Besides, therapy and treatment monitoring could be a feasible end-point for such technology [3]. It is worth remarking that in this preliminary phase the use of innovative techniques must be supported by consolidated procedures and instruments [4]. In the case of halitosis the OralChroma™ device and panellists are considered the standard [5]. This research has been designed with the objective of monitoring disease treatment and testing by using a recently developed multi-sensor approach, called BIONOTE^®^.

In order to test the ability of this system to monitor halitosis treatment, *Lactobacillus brevis* (CD2)–containing lozenges were proposed to be tested, as a safe and effective treatment for periodontal diseases for various reasons, highlighted below [6].

The possible utility of *Lactobacillus brevis* (CD2) in the treatment of halitosis derives from arginine deiminase (AD), which has effects against halitosis by inhibiting the formation of volatile sulfur compounds (VSC) in the oral cavity. More specifically, *Lactobacillus brevis* (CD2) is able, through its arginine-deiminase activity, to removing the substrate (arginine) of nitric oxide (NO) synthase and polyamines (putrescine and spermidine). The inhibition of the formation of NO causes a significant inhibition of synthesis of prostaglandins, especially under inflammatory conditions, and blocks the increase in the production of proteolytic enzymes. This will limit the sources of peptides and amino acids for odorous bacteria, with the result of less formation of VSC and polyamines. The reduced amount of such cytotoxic agents facilitates ambient conditions more compatible with a good oral health status. Moreover, the limitation of the nutritional substances makes the growth of bacteria more difficult. In addition, the reduced formation of NO via the AD may counteract the inhibitory effect of lipopolysaccharide (LPS) of Gram-negative bacteria on the synthesis of salivary mucin, and the protective role of salivary mucin is known [7,8].

Finally, the role of arginine to make bacteria resistant to acids, so that they can survive in an environment with variable acidity is well known. The depletion of the extracellular arginine by *Lactobacillus brevis* (CD2) could counteract the protective acidic neutralization of the bacteria, thus making them less resistant to changes in acidity [9].

The aim of this randomized clinical trial is to evaluate whether the recently described multi-sensor approach called BIONOTE^®^ is accurate enough to verify the efficacy of treatment of patients with halitosis using *Lactobacillus brevis* (CD2)–containing lozenges, comparing the data with patients treated with placebo. The recently developed technique was tested through the comparison with traditional techniques: OralChroma™ and two calibrated odor judges enrolled for the organoleptic assessments.

## 2. Experimental Section

### 2.1. Trial Design

This is a monocenter, randomized, double-blind, placebo-controlled parallel-group study conducted in Italy. No changes to methods were done after trial commencement.

### 2.2. Participants

Possible patients for enrollment in the study were recruited by means of public posters and brochures in both the dental clinic of the University of L’Aquila, and pharmacies in the city of L’Aquila. Twenty patients were included in the study (10 treated with *Lactobacillus brevis* (CD2)–containing lozenges and 10 with placebo) suffering from active phase halitosis.

All the subjects who showed up for possible enrollment in the study were provided a free dental visit.

The eligibility criteria were:

(a) Adult age (>18 years of age);
(b) Halitosis in active phase;
(c) Informed consent by the patient.

Exclusion criteria were:

(a) The need to take antibiotics for the presence of signs and/or symptoms of infection;
(b) Use of non-steroidal anti-inflammatory drugs during the 30 days prior to the beginning of the study;
(c) Use of steroid medications during the 30 days prior to the beginning of the study;
(e) Dental care in progress;
(f) Current gingivitis and periodontitis;
(g) Systemic diseases such as: chronic liver disease, chronic renal failure, gastro-esophageal reflux;
(h) Alcoholism and/or drug addiction.

The coexistence of other diseases was allowed, with the exception of those enumerated in the exclusion criteria. Informed consent was obtained from each patient included in the study.

### 2.3. Study Setting

The study took place at the Research Centre for the Diagnosis and Treatment of Halitosis, University of L’Aquila (L’Aquila, Italy) from January 2014 to June 2014. The Ethics Committee of the University of L’Aquila approved the protocol.

### 2.4. Interventions

Patients received from the principal investigator (E.M.) three boxes, each containing 20 tablets (*Lactobacillus brevis* CD2—containing lozenges or matching placebo).

The day after, patients started taking *Lactobacillus brevis* (CD2)–containing lozenges or placebo every day for 14 days, 4 tablets/day. With regard to additional therapies, if comorbidities were present, the use of other drugs was allowed, provided that—according to the investigator—this did not alter, in a significant manner, the patient’s response to the treatment under study, and/or did not fall within the exclusion criteria listed in the Protocol. The principle investigator (E.M.) provided an explanation to each patient of the characteristics and the mode of administration of the drug.

The duration of the trial was established to be 14 days. At the end of this period, the experimenter (E.M.) provided the coordinator of the study (G.M.) with a report including data about the conduct and results of the trial. For the outcomes analysis, t_0_ was before the beginning of the study; t_1_ was day 7 and t_2_ was day 14. At t_0_, t_1_ and t_2_, the assessment of outcome measures was carried out. 

**Figure 1:**
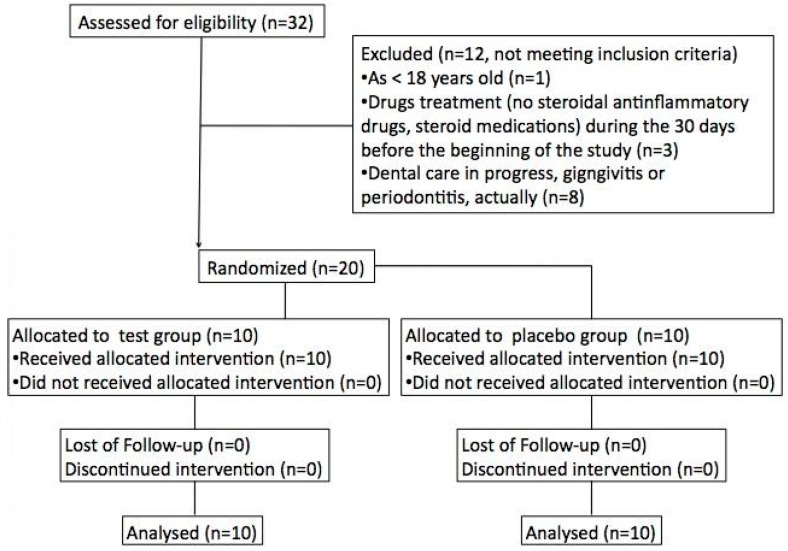
Flow diagram of the CONSORT 2010 study.

The selection procedure from baseline screening to enrollment is described in Figure 1.

### 2.5. Outcomes

The primary endpoint with respect to efficacy in halitosis is the proportion of patients achieving an improvement in outcome measures from baseline (t_0_) to day 7 (t_1_) and day 14 (t_2_).

Outcome measures are:

- the Breathprint (BP) obtained with the recently introduced technique, called BIONOTE^®^ (at t_0_, t_1_ and t_2_) (this is the “test technique”, described above);
- the Rosenberg score [10], an organoleptic measurement assessed by two calibrated odor judges enrolled for the study, which is, at the actual state of the art, a gold standard, apart from the gas chromatography, in the diagnosis of halitosis;
- the Winkel Tongue Coating Index, anterior and posterior (WTCI, anterior and posterior) [11] an index to evaluate the tongue coating, assessed by two calibrated operators enrolled for the study;
- the gas chromatography score (OralChroma™) [12].

No changes to trial outcomes after the trial commencement were decided. Additional analyses are done on the correlations among the different methods in targeting halitosis.

The recording of outcomes measurements began with the OralChroma™ because this tool takes 8 min for each analysis. Meanwhile the breath samples were collected for the BIONOTE^®^ analysis. Finally, the organoleptic Rosenberg score and the WTCI score (anterior and posterior) were collected.

The day before the evaluation, the subject were forbidden to consume alcohol or smoke. Patients were asked not to eat, drink, smoke, nor perform oral hygiene for at least one hour before their appointment. Before the breath collection procedure, all patients were requested to rinse their mouth, without using toothpaste. During the assessment of the outcome measurements, the subjects avoided any rinsing between the collections. Globally, the assessment lasted about 10 min. The outcome measures and how they were assessed are summarized in Table 1.

**Table 1:** Outcome measures and description of how they were assessed.

Outcome Measures	Description of How Outcome Measures Were Assessed
Winkel Tongue Coating Index (WTCI), anterior and posterior	The dorsum of the tongue was divided into six areas (three posterior, three anterior) and tongue coating was assessed in each sextant as follows; 0 = no coating, 1 = light coating, 2 = severe coating. The WTCI was obtained by adding all six scores, for a possible range of 0–6 for WTCI anterior and 0–6 for WTCI posterior.
The Rosenberg score, organoleptic measurement	The organoleptic measurement depends on a trained examiner that has demonstrated reliability in smelling halitosis. The operator, preferably blindfolded, sniffs the air exhaled from the mouth at a distance of 10 cm. For recording a scale of 5 values is used. The test is considered positive when the “hedonic” value assigned to breath exceeds the number 2. The scale includes the following values: 0 = no odor; 1 = doubtful presence of halitosis; 2 = slight odor but clearly notifiable; 3 = moderate halitosis; 4 = strong halitosis; 5 = very intense halitosis.
Gas chromatography score, measured with OralChroma™	Measure the molecular levels of the three major VSCs (hydrogen sulphide, methyl mercaptan and dimethyl sulfide) in a sample of mouth air. The levels (measured in ppm) are reported in a diagram from low to high level. A cognitive threshold is individuated and levels are individuated as “more than” or “less than” the cognitive threshold.
BP (breath print) constructed by BIONOTE^®^	Individual Breathprint (BP) of a patient is represented with a radar plot; equiangular radii shape each radar plot, where each radius represents one of the 28 sensor responses. The radius length gives magnitude of each sensor response (expressed in Hz, because relative to a resonant frequency shoft of the quartz slice). The radar plot “profile” consists of a line drawn connecting the data values for each radius.

The BIONOTE^®^ technique has been described elsewhere [13]. Its characteristics can be thus summarized: the BIONOTE^®^ (for BIOsensor-based multisensorial system for mimicking Nose, Tongue and Eyes) is a multisensory system recently introduced by two of the authors (G.P. and M.S.), able to analyse the volatile and liquid parts of a sample. In this clinical trial, only the gas sensor array is employed. The transducers used for the gas sensor array are seven quartz crystals with a resonance frequency of 20 MHz in the thickness shear mode covered with a combination of anthocyanins extracted by three different plant tissues: red rose, red cabbage, and blue hortensia [14].

**Figure 2:**
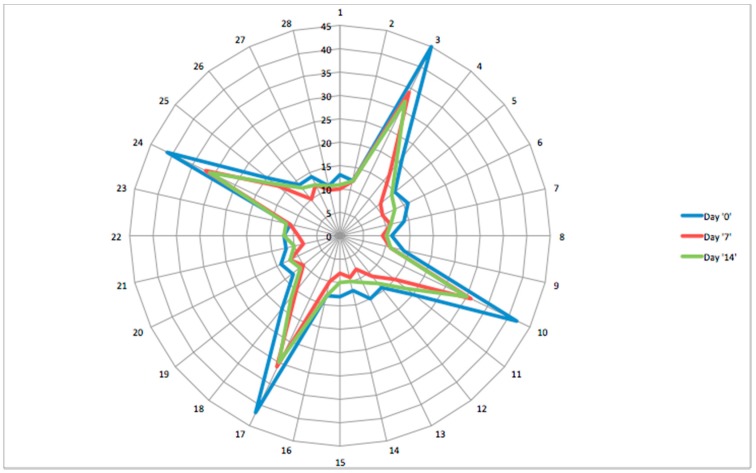
The Breathprint (BP) of one of the patients at day 0, day 7 and day 14.

The final dataset of the gas analysis is composed of 28 responses due to the registration of the seven sensors’ behaviour at four different temperatures of the sample (this procedure is dependent on the exhaled breath sampling on the adsorbent cartridge). The desorption of the cartridge into the sensors chamber is obtained by an interfacing device able to uniformly heat the tube from 50 to 200 °C, and finally cleaning the cartridge holding the temperature at 300 °C for 5 min. The final fingerprint of the exhaled breath is a sequence of four n-dimensional patterns, composed of the n responses of an n-dimensional gas sensor array at four temperatures (50–100–150–200 °C). The individual Breathprint (BP, Figure 2) of a patient is represented with a radar plot; equiangular radii shape each radar plot, where each radius represents one of the 28 sensor responses. The radius length gives the magnitude of each sensor response. The radar plot “profile” consists of a drawn line connecting the data values for each radius.

### 2.6. Breath Collection

Breath collection for the BIONOTE^®^ analysis is performed through a procedure recently introduced by two of the authors (G.P. and M.S.) [15,16]. Each patient was asked to breathe for 3-min at tidal volume into a dedicated storage device for direct sampling of exhaled breath on an adsorbing cartridge [14]. The adsorbent cartridge used in this work was a Tenax GR by Supelco [17]. The samples (adsorbent cartridges) were then transported from L’Aquila to the Centre for Integrated Research—CIR, Unit of Electronics for Sensor Systems, University Campus Bio-Medico (Rome, Italy) where the BIONOTE™ device is located.

No interim analyses were performed as the trial lasted 14 days. The stopping guidelines established that if a patient experienced side effects, a delay taking the study drug was planned. 

### 2.7. Randomisation, Allocation and Blinding

Participants were randomly assigned to treatment or placebo groups following simple randomization procedures (computerized random numbers). The allocation of treatment or placebo group was undertaken by a person not directly involved in the research project. The operator assessing outcomes and data collectors were blinded to the allocation of subjects.

*Lactobacillus brevis* (CD2)–containing lozanges and matched placebo were in lozenge form and had identical appearance. They were prepacked in boxes, each containing 20 tablets, with the same look and the same weight, so it was impossible to distinguish them *a priori*. The packages were consecutively numbered according to the randomisation schedule. Each participant was assigned an order number and received three boxes, each containing 20 tablets.

### 2.8. Statistical Methods

#### 2.8.1. Adverse Events

The evaluation of the tolerability of *Lactobacillus brevis* (CD2) was based on the incidence and type of adverse events. Particular attention was given to any undesired event occurred during the 14 day testing period, reporting:

(a) Type of event;
(b) Characteristics of the undesired event;
(c) Date of onset;
(d) Duration (including whether it is expressed in minutes, hours or days);
(e) Maximum intensity reached;
(f) Mode of onset (immediate, gradual or asymptomatic);
(g) Possible therapy of the undesired event;
(i) Report of causality with the product;
(l) Outcome of the undesired event: if it disappeared without modification of the treatment or after stopping the treatment, or if it remained but the study could be continued.

#### 2.8.2. Effectiveness of the Treatment

The assessment of the effectiveness of the treatment was carried out through with a multiparametric approach: (1) the parameters measured via the panellist assessment (Rosenberg score and WTCI, anterior and posterior); (2) the OralChroma™; (3) the BIONOTE^®^ (test analysis).

The data analysis has been conducted following this flow-chart:

(1) Treatment efficacy assessment via each single technique:
  - By ANOVA test for panellist analysis: Rosenberg score, WTCI (anterior) and WTCI (posterior) (t_0_, t_1_ and t_2_ assessments)
  - By partial least square—discriminate analysis (PLS-DA) for OralChroma™
  - By PLS-DA for BIONOTE^®^
(2) Treatment efficacy assessment via a data fusion of the three techniques’ outputs using PLS-DA
(3) Correlation study between the three techniques

Partial least square discriminate analysis (PLS-DA) has been applied, with leave-one-out as cross-validation. In the case of small populations, in fact, the leave-one-out is a good cross-validation criterion using all the measurements of the dataset as training and test samples. PLS-DA was used as a regression method for calculating to which extent basal data could predict post-treatment data, and the differences between the study and the placebo groups. PLS-DA has been performed using the PLS-Toolbox SW (Eigenvector, Wenatchee, WA, USA) in the MATLAB environment (The Mathworks, Natick, MA, USA). A data pre-treatment was necessary for both data fusion and mapping, in order to overcome different dimensionalities of the data-sets: a linear normalization procedure has been used to this purpose.

## 3. Results and Discussion

### 3.1. Results

#### 3.1.1. Participant Flow

No subject losses were registered. Eligible participants were recruited from January 2014 to June 2014. Participants attended clinic visits at the time of randomisation (baseline, t_0_) and at 7-days intervals for 14 days. Baseline demographic data are reported in Table 2. The analysis involved all patients who were randomly assigned.

**Table 2:** Baseline demographic data.

	*Lactobacillus brevis* (CD2)—Containing Lozenges (*n* = 10)	Placebo (*n* = 10)
**Age (mean years ± SD)**	33 ± 9	36 ± 7
**Sex (males)**	12	11

#### 3.1.2. Outcomes and Estimation

(a) Treatment efficacy assessment with the different scores

The analysis only provided statistically significant differences over time for the test group treated with *Lactobacillus brevis* (CD2)–containing lozenges for the WTCI anterior test, and non-significant changes over time for the placebo group.

Specifically:

When the Rosenberg scores were analyzed, the ANOVA analysis revealed non-statistically significant changes over the time both for the placebo group (*p* = 0.074) and for the test group (*p* = 0.24). The same was true for the WTCI posterior test, for which the ANOVA analysis revealed non-statistically significant changes over time both for the placebo group (*p* = 0.074) and for the test group (*p* = 0.14).

When the WCTI anterior data was analyzed, the ANOVA analysis revealed a statistically significant change over the time (t_0_, t_1_ and t_2_; *p* = 0.037) for the test group; *post-hoc* analysis revealed statistically significant changes between t_0_ and t_2_ data (*p* = 0.014).

**Figure 3:**
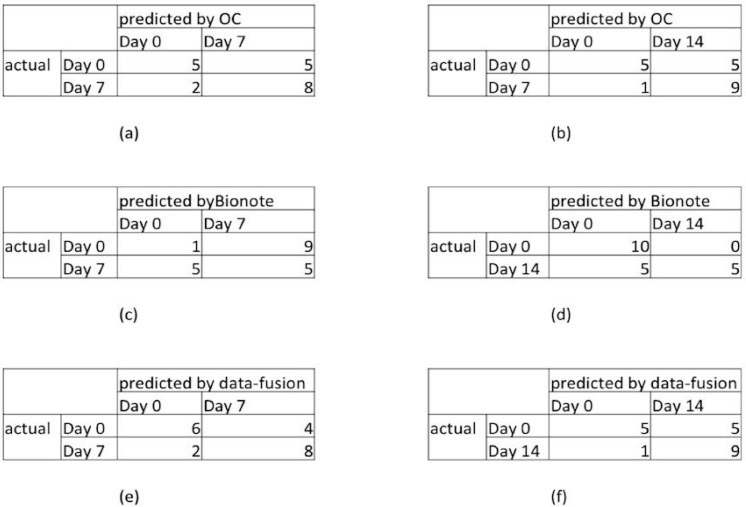
Results of the experiment. Predicted cases at Day 0 (t_0_), Dat 7 (t_1_) and Day 14 (t_2_). OC: OralChroma™; (**a**,**b**) predicted by OralChroma™; (**c**,**d**) predicted by BIONOTE^®^; (**e**,**f**) predicted by data fusion. See text for details.

The PLS-DA model calculated on the OralChroma™ data showed different performances when used in different binary problems:

- Binary classification (0–7): correct classification of 50% of day 0 and of 80% of day 7 (see the confusion matrix reported in Figure 3a).
- Binary classification (0–14): correct classification of 50% of day 0 and of 90% of day 14 (see the confusion matrix reported in Figure 3b).
- Binary classification (7–14): non-significant results.

For the data obtained through the BIONOTE^®^ the basal BPs of the 40 patients suffering from halitosis appeared heterogeneous in profile and intensity. In addition, the visual analysis of radar plots showed directional change in BPs after treatment in the test group. The PLS-DA model calculated on the BIONOTE^®^ data showed different performance when used in different binary problems:

- Binary classification (0–7): correct classification of 90% of day 0 and of 50% of day 7 (see the confusion matrix reported in Figure 3c).
- Binary classification (0–14): correct classification of 100% of day 0, and of 50% of day 14 (see confusion matrix reported in Figure 3d).
- Binary classification (7–14): not significant results.

(b) Treatment efficacy assessment via a data fusion of the three technique

When data fusion of the three techniques’ outputs using the PLS-DA was analyzed, the PLS-DA model calculated on the data fusion of BIONOTE^®^ and OralChroma™ outputs showed different trends in different problems. Specifically:

- Binary classification (t_0_–t_1_): correlated classification in 60% of cases at t_0_, and 80% at t_1_ (see the confusion matrix reported in Figure 3e);
- Binary classification (t_0_–t_2_): correlated classification in 50% of cases at t_0_, and 90% at t_2_ (see the confusion matrix reported in Figure 3f);
- Binary classification (t_1_–t_2_): non-significant results.

#### 3.1.3. Correlation Study between the Three Techniques

The BIONOTE^®^ data allowed us to predict WCTI anterior, WCTI posterior and Rosenberg scores with a *root mean square error in cross-validation* (RMSeCV) of 0.52, 0.73 and 0.78, respectively. The correlation between BIONOTE^®^ and methylmercaptan (CH_3_SH) was RMSeCV = 111. The correlation between OralChroma™ and other techniques showed a rho <0.25. No adverse effects were registered.

Data analysis was performed in the MATLAB environment, by means of the PLS Toolbox. A partial least square discriminant analysis (PLS-DA) was performed, considering two classes in each model. In order to evaluate the identification performances of the method, a leave-one-out validation criterion has been adopted. 

**Table 3:** Oral Chroma™.

	RMSEC LV1	RMSEC LV2	RMSECV LV1	RMSECV LV2	#LV	LV1 PRESS	LV2 PRESS
Binary classification (0–7)	0.45	0.43	0.49	0.48	2	4.88	4.74
Binary classification (0–14)	0.46	0.45	0.542	0.541	2	5.89	5.87
Binary classification (7–14)	0.44	0.43	0.47	0.49	2	6.7	7.2

**Table 4:** BIONOTE^®^.

	RMSEC LV1	RMSEC LV2	RMSECV LV1	RMSECV LV2	#LV	LV1 PRESS	LV2 PRESS
Binary classification (0–7)	0.43	0.41	0.45	0.44	2	4.75	4.66
Binary classification (0–14)	0.44	0.43	0.47	0.46	2	4.89	4.75
Binary classification (7–14)	0.56	0.54	0.51	0.54	2	6.4	7.1

**Table 5:** Data fusion.

	RMSEC LV1	RMSEC LV2	RMSECV LV1	RMSECV LV2	#LV	LV1 PRESS	LV2 PRESS
Binary classification (0–7)	0.61	0.54	0.50	0.51	2	8.77	6.43
Binary classification (0–14)	0.60	0.58	0.66	0.59	2	8.79	7.11
Binary classification (7–14)	0.53	0.50	0.55	0.53	2	8.32	8.58

Model parameters are reported above. Each parameter is extracted from the model. The number of latent variables is selected considering a decrease of RMSEC, RMSECV and the PRESS. In particular the PRESS of binary classifications (7–14) increase for the second latent variable in all cases which a typical condition of overfitting (Table 3, Table 4 and Table 5).

### 3.2. Discussion

The short follow-up duration is a limitation of our study. As the participants were recruited among subjects who voluntarily answered the call that took place through brochures—so presumably the subjects that responded had a psychological interest in addressing the problem of halitosis—the present findings seem to be applicable for this class of subjects.

The primary outcome of this study was the evaluation of the accuracy of a recently developed system—the BIONOTE^®^—in the assessment of treatment outcomes, in comparison with traditional systems, such as OralChroma™, and two calibrated odor judges enrolled for the organoleptic assessments (a).

In addition, the efficacy of *Lactobacillus brevis* (CD2)–containing lozenges in the treatment of halitosis, comparing data with a placebo, was evaluated (b).

(a) The evaluation of the accuracy of a recently developed system—the BIONOTE^®^—in the assessment of treatment outcomes

When the accuracy of the methods was assessed via a data fusion of the techniques, the results suggested a proper utilization of the BIONOTE^®^ in addition to OralChroma™ consisting of their combination to assess the initial condition of halitosis (diagnosis at t_0_) by BIONOTE^®^ and its mitigation during treatment by OralChroma™.

The good results obtained by data fusion (Figure 3e,f) suggested verifying whether these multidimensional systems could predict the traditional panellist indexes. BIONOTE^®^ data allowed us to predict WTCI anterior, WCTI posterior and Rosenberg scores, with a *root mean square error in cross-validation* (RMSeCV) of 0.52, 0.73 and 0.78, respectively, while the correlation between OralChroma™ and panellists’ indexes showed a rho < 0.25.

(b) Efficacy of *Lactobacillus brevis* (CD2)—containing lozenges in the treatment of halitosis

The WTCI anterior showed statistically significant changes between t_0_ and t_2_ (*p* = 0.014) in the treated group. These results suggest a significant effect of *Lactobacillus brevis* (CD2)–containing lozenges in reducing tongue coating and the total number of bacteria (mostly *Fusobacterium nucleatum*) on the dorsal surface of the tongue responsible for VSC.

A possible explanation could be that *Lactobacillus brevis* (CD2) can reverse (or attenuate) the inhibitory effect of LPS on the formation of mucin, so restoring the “washing” effects of mucin in this area. Another explanation could be that with the increase of the extracellular pH due to the introduction of AD during therapy, *Fusobacterium nucleatum* increases the use of glucose [18], instead of aminoacids [19], with a consequent reduction of the formation of VSC from *Fusobacterium nucleatum* on the dorsal suface of tongue. In addition, the odorous bacteria on the dorsal surface of the tongue produce methyl mercaptan from methionine, using arginine [20]. The reduction, by CD2, of the substrate (arginine) can consequently reduce intracellular polyamines—such as putrescine, spermidine and spermine [21,22].

Apart from WTCI, the other methods did not evidence statistically significant changes over the duration of the treatment. Consequently, the tested treatment only showed a significant efficacy in the treatment of the dorsal surface of the tongue. The study failed to evidence a statistically significant effect of the tested treatment on halitosis after 14 days of treatment. Long-term efficacy of this treatment cannot be excluded, also considering the small sample of this study, and the fact the initial halitosis values were not very serious. 

## 4. Conclusions

The aim of this randomized clinical trial was to validate a new multi-sensor approach called BIONOTE^®^ in the monitoring of treatment of halitosis, in comparison with traditional techniques, and to evaluate the clinical efficacy of *Lactobacillus brevis* (CD2)–containing lozenges in the treatment of halitosis. The multisensor system BIONOTE^®^ can be used in addition to OralChroma™ to assess the initial condition of halitosis (diagnosis at t_0_) by BIONOTE^®^ and its mitigation during treatment by OralChroma™.

The study failed to evidence a statistically significant efficacy of *Lactobacillus brevis* (CD2)–containing lozenges in the treatment of halitosis after 14 days, although a statistically significant reduction of tongue coating on the dorsal surface of tongue (WTCI anterior) was observed in the test groups, compared to the placebo group. Long-term efficacy of the treatment cannot be excluded, as this trial only lasted 14 days.
